# Perinatal fluoxetine exposure disrupts the circadian response to a phase-shifting challenge in female rats

**DOI:** 10.1007/s00213-020-05556-2

**Published:** 2020-06-12

**Authors:** Danielle J. Houwing, Jolien de Waard, Anouschka S. Ramsteijn, Tom Woelders, Sietse F. de Boer, Emma J. Wams, Jocelien D. A. Olivier

**Affiliations:** 1 Department of Neurobiology, unit Behavioral Neuroscience, GELIFES, University of Groningen, Nijenborgh 7, 9747 AG Groningen, the Netherlands; 2 Department of Neurobiology, unit Chronobiology, GELIFES, Univ. Groningen, Nijenborgh 7, 9747 AG Groningen, the Netherlands

**Keywords:** Fluoxetine, Pregnancy, Perinatal, Circadian behavior, Anxiety, Coping style, Clock genes, 5-HT1A receptor, Hypothermia

## Abstract

**Rationale:**

Selective serotonin reuptake inhibitor (SSRI) antidepressants are increasingly prescribed during pregnancy. Changes in serotonergic signaling during human fetal development have been associated with changes in brain development and with changes in affective behavior in adulthood. The suprachiasmatic nucleus (SCN) is known to be modulated by serotonin and it is therefore assumed that SSRIs may affect circadian rhythms. However, effects of perinatal SSRI treatment on circadian system functioning in the offspring are largely unknown.

**Objective:**

Our aim was to investigate the effects of perinatal exposure to the SSRI fluoxetine (FLX) on circadian behavior, affective behavior, and 5-HT_1A_ receptor sensitivity in female rats. In addition, we studied the expression of clock genes and the 5-HT_1A_ receptor in the SCN, as they are potentially involved in underlying mechanisms contributing to changes in circadian rhythms.

**Results:**

Perinatal FLX exposure shortened the free-running tau in response to the 5-HT_1A/7_ agonist 8-OH-DPAT. However, FLX exposure did not alter anxiety, stress coping, and 5-HT_1A_ receptor sensitivity. No differences were found in 5-HT_1A_ receptor and clock genes Per1, Per2, Cry1, and Cry2 SCN gene expression.

**Conclusions:**

Perinatal FLX exposure altered the response to a phase-shifting challenge in female rats, whether this may pose health risks remains to be investigated.

## Introduction

Depressive symptoms occur frequently during pregnancy, resulting in a major depressive disorder (MDD) in about 5% of pregnant women (Gaynes et al. 2005; Melville et al. 2010). Sometimes antidepressant treatment is unavoidable, especially when a mother is suicidal. In Europe, about 3% of pregnant women are prescribed antidepressants, while in the USA, numbers rise up to 13% (Cooper et al. 2007; El Marroun et al. 2012; Hayes et al. 2012). Selective serotonin reuptake inhibitors (SSRIs) are the first-line treatment for MDD during pregnancy and lactation, as they have not been associated with increased risks for structural teratogenic effects in the offspring (Gentile 2005). However, SSRIs do reach the developing child through the placenta and are evident in breast milk (Heikkinen et al. 2003; Noorlander et al. 2008). SSRIs operate by blocking the serotonin (5-HT) transporter and inhibiting 5-HT reuptake back into the presynaptic cell. At first, the increased 5-HT levels act on the more distal 5-HT_1A_ autoreceptors inhibiting further neuronal firing, but chronic use of SSRIs results in downregulation of these 5-HT_1A_ receptors. The removal of this 5-HT_1A_ receptor-mediated negative feedback loop ultimately results in sustained higher extracellular 5-HT levels and increased serotonergic neurotransmission (Pierz and Thase 2014). During development, serotonin acts as a neurotrophic factor regulating various cell processes such as cell division, cell differentiation, synaptogenesis, neurite sprouting, and dendritic pruning (Gaspar et al. 2003). Altering serotonin levels through SSRI exposure during development can therefore lead to changes in brain development and adult behavior (reviewed in (Olivier et al. 2013)). For example, internalizing and externalizing behaviors, as well as anxiety and depression, are more often observed in children of women who used SSRIs during the perinatal period (Hanley et al. 2013, 2015; Brandlistuen et al. 2015; Malm et al. 2016), while other studies show that not the SSRIs but the maternal mood during the postpartum period is responsible for these outcomes (Oberlander et al. 2007; Oberlander, T. F., Papsdorf, M., Brain, U.M., Misri, S., Ross, C. and Grunau 2010). Similarly, effects of perinatal SSRI treatment on neurodevelopment, serotonergic functioning, and adult behavior are found in rodent studies. For example, perinatal exposure to the SSRI fluoxetine (FLX) has been associated with increased 5-HT_1A_ receptor sensitivity (Olivier et al. 2011; Altieri et al. 2015) and higher levels of anxiety in rodent offspring (Olivier et al. 2011; Ko et al. 2014; Boulle et al. 2016a). Furthermore, SSRIs are known to influence the sensitivity to the circadian system (McGlashan et al. 2018). However, effects of maternal SSRI treatment during the perinatal period on circadian system functioning in the offspring are largely unknown. The suprachiasmatic nucleus (SCN) of the hypothalamus is also known as the master pacemaker of circadian rhythms, which drives daily essential functions of the body such as feeding, sleeping, body temperature, and the secretion of hormones by synchronizing the internal environment with the external environment (Gillette and Tischkau 1999). These functions repeat themselves over a period of near 24 h and are termed circadian rhythms. The period of the circadian rhythm in constant conditions (such as constant darkness) is called the free-running period, or tau. Furthermore, even though circadian rhythms are self-sustaining, they rely upon circadian time cues (zeitgebers), which can be either photic (light) or nonphotic (e.g., activity, temperature), to adjust to the local environment. Furthermore, circadian rhythms are generated and regulated by highly conserved “clock” genes that are expressed in the SCN, with oscillating mRNA and protein levels of near 24 h, with Period 1 (Per1), Period 2 (Per2), Cryptochrome 1 (Cry1) and Cryptochrome 2 (Cry2) being among some of the first identified clock genes. Interestingly, SSRI exposure in adulthood has been found to shorten expression of Per1 in rat-1 fibroblasts and in the mouse SCN (Nomura et al. 2008), thus affecting circadian rhythmicity. The SCN mainly receives serotonergic input from the median raphe nuclei and in turn indirectly projects to midbrain raphe nuclei (Morin 2013). In the SCN, serotonergic signaling can modulate phase-shifting effects of photic and nonphotic cues both in vitro (Medanic and Gillette 1992; Prosser 2003; Prosser et al. 2006) and in vivo (Ehlen et al. 2001; Smith et al. 2008). In addition, 8-OH-DPAT, a 5-HT_1A/7_ receptor agonist, is well known as a nonphotic stimulus to induce phase advances in circadian rhythm (partly) via the 5-HT_1A_ receptor (Smith et al. 2008). Interestingly, SSRIs alter the sensitivity of 5-HT receptors, mostly reducing the sensitivity of the 5-HT_1A_ receptor (Olivier et al. 2011). It is therefore assumed that SSRI exposure, e.g., during development, also affects circadian rhythms. Indeed, when male mice are exposed to FLX during the perinatal period, they show a shorter free-running period in total darkness, an increased phase shift to photic cues, and a decreased phase shift to nonphotic cues, suggesting persistent changes in the circadian system (Kiryanova et al. 2013, 2017). Despite behavioral alterations being observed, the mechanisms involved were not explored in these studies. One of the possible mechanisms through which the alterations in circadian rhythm caused by SSRIs take place might be the 5-HT_1A_ receptor. Both presynaptic and postsynaptic 5-HT_1A_ receptors exist. Presynaptic 5-HT_1A_ autoreceptors are present on soma and dendrites of the raphé serotonergic neurons projecting to many forebrain areas (Fernández-Guasti et al. 1992; Le Poul et al. 1995; Marek 2010; Altieri et al. 2013). Postsynaptic 5-HT_1A_ heteroreceptors are present in various brain areas, mainly in the forebrain (Frink et al. 1996; Garcia-Garcia et al. 2017). Postsynaptic 5-HT_1A_ receptors are implicated in mood disorders and have been used as target for various neuropsychiatric disorders (Newman-Tancredi 2011). Genetic studies in both 5-HT_1A_ receptor knockout mice and humans indicate that the 5-HT_1A_ receptor may be an underlying mechanism in major depressive disorders (Savitz et al. 2009) and anxiety disorders (Lesch et al. 1992; Olivier et al. 2001; Nash et al. 2008). In humans, changes in circadian behavior can result in altered physiological responses and changes in sleep-wake behavior which can contribute to disease pathology (Smolensky et al. 2016). In this study, our aim was to investigate the effects of perinatal FLX treatment on (1) circadian rhythms for both body temperature and activity, including phase shifts in response to photic (light) and nonphotic cues such as the 5-HT_1A/7_ agonist 8-OH-DPAT, (2) the sensitivity to 5-HT_1A_ receptor agonist induced hypothermia, (3) anxiety and stress coping behavior, and (4) gene expression levels of the 5-HT_1A_ receptor and clock genes in the SCN. Because circadian parameters may be linked to depression in different ways in males and females (Swanson et al. 2017), and a higher prevalence of affective disorders is found in women compared to men, our aim was to understand the effects of perinatal FLX exposure in female rats.

## Materials and methods

### Animals and housing

Animals were housed in Makrolon type 3 cages (38.2 × 22.0 × 15.0 cm) during individual housing or Makrolon type 4 cages (55.6 × 33.4 × 19.5 cm) during social housing. Animals had ad libitum access to food (RMH-B, AB Diets; Woerden, the Netherlands) and tap water and were maintained on a reversed 12 h light/dark cycle (lights off at 11:00 A.M.) unless stated otherwise. A wooden gnawing stick (10 × 2 × 2 cm) and nesting material (Enviro-dri®) was provided for environmental enrichment. All breeding took place in our own facility and experimental procedures were approved by the Groningen University Committee of Animal experiments.

### Breeding

Animals were surplus animals from a previous experiment (Houwing et al. 2019b) where female heterozygous serotonin transporter (SERT) knockout rats (Slc6a41Hubr; SERT^+/-^) were bred with SERT^+/−^ male rats. Wildtype (SERT^+/+^), SERT^+/-^, and homozygous SERT knockout (SERT^−/−^) pups were exposed to early life stress (ELS) by being maternally separated for 6 h a day, while control (CTR) pups were separated and handled for 15 min, daily from postnatal day (PND) 2–15. Pups were weaned at PND21 and socially housed with same-treated, same-sex pups from different litters. When adult, ELS SERT^+/-^ female pups showed depressive-like behavior and were used as an animal model for maternal depression (Houwing et al. 2019a). However, in the present study, we used only wildtype (SERT^+/+^) female offspring from the nondepressive CTR SERT^+/^^-^ dams (with and without FLX treatment), as the offspring from these dams were surplus.

### Perinatal FLX treatment

Wistar CTR SERT^+/-^ females were bred with SERT^+/+^ males when in estrus, which was determined by measuring vaginal wall impedance (model MK-11, Muromachi, Tokyo, Japan). Females were housed with males for 24 h, and this day was set as gestational day 0 (G0). Dams were randomly assigned to the vehicle (VEH) or FLX-treated group. FLX (2 mg/mL; Pharmachemie BV, the Netherlands) or VEH (methylcellulose 1%, Sigma Aldrich Chemie BV, Zwijndrecht, the Netherlands) was dissolved in sterile water and administered at a volume of 0.5 mL/100 g using oral gavage (PVC flexible feeding tubes, Vygon, Valkenswaard, the Netherlands). Dams were treated daily with 10 mg/kg FLX or VEH from G0 until weaning of the pups at PND21. During oral gavage, animals were held but not restrained, minimizing stress. To determine the exact injection volume, dams were weighed daily. Dams were checked twice a day for pup delivery. On PND21, pups were weaned and housed with 3–5 same-treated, same-sex animals, from different litters. Ears were punched for individual recognition and genotyping (for genotyping protocol, see (El Aidy et al. 2017)). Only SERT^+/+^ females were used for the current study.

### Behavioral testing

Adult female wildtype (SERT^+/+^) offspring (12 VEH, 11 FLX) were tested for anxiety and stress coping using various behavioral tests. Naïve animals were first tested in the elevated plus maze (EPM) and the forced swim test (FST). At the end of all other experiments, animals were tested in the home cage emergence test (HCE) and again in the EPM. Testing occurred between 12:00 and 17:00 during the dark phase of the animals, under dim light conditions. Before testing, the estrous stage of each animal was measured using a vaginal wall impedance checker (model MK-11, Muromachi, Tokyo, Japan). Animals were only tested when not in estrous. Animals were socially housed (3–4 per cage) during the first EPM and the FST, and individually housed when the HCE and the second EPM were performed.

#### Elevated plus maze

At 16 weeks of age, animals were tested for anxiety-like behavior in the elevated plus maze (EPM). In short, the EPM consisted of two open (45.0 × 10.0 × 1.0 cm) and two closed (45.0 × 10.0 × 50.0 cm) arms located opposite of each other. The EPM was elevated at a height of 50 cm. Rats were placed in the center of the plus maze facing an open arm and were allowed to freely explore for 5 min. Frequency and time spent on the open arms and distance moved on the open arms were automatically scored using EthoVision XT 12.0 (Noldus Information Technology B.V., Wageningen, the Netherlands). Animals were tested under dim light conditions (open arm 2.5 lx; closed arm 0.25 lx) and the EPM was cleaned with 70% ethanol between trials. Animals were tested in the EPM again at 41 weeks of age, after ending of all pharmacological challenges.

#### Forced swim test

At 17 weeks of age, animals were tested for their stress coping style in the forced swim test (FST). Animals were placed in cylindrical Plexiglas tanks (50.0 × 18.0 cm diameter), which were filled with 30 cm of water (22 ± 1 °C). On the first day, 3 or 4 animals were placed in the separate tanks for 15 min. Animals were dried with a towel and placed in a cage on a heating mat to recover while the next trial commenced. On day 2, exactly 24 h later, animals were re-tested for 5 min. Animals could not see each other as tanks were separated with dividers. Animals were not tested if they were in estrus on the first day, but we did not take the estrus cycle into account on day 2. The FST was recorded on video camera and the duration of mobility and immobility on day 2 was scored by an observer blind for treatment using The Observer XT 11.0 (Noldus Information Technology B.V., Wageningen, the Netherlands). Active climbing, swimming, and diving were scored as mobility, while immobility was defined as making no movements for at least 2 s or making only those movements that were necessary to keep the nose above the water. Slightly moving paws or support by pressing paws against the wall of the tank was still considered immobility. Tanks were cleaned and refilled between trials.

#### Home cage emergence

At 41 weeks of age, after all other challenges were completed, animals underwent the home cage emergence test, which is another test for anxiety-like behavior. The animal, which was individually housed in its home cage, was moved to an adjacent test room and placed in an open field (100 × 100 cm). The lid of the cage was removed and a wired metal grid was placed over the edge of the cage for easier escape. Using a stopwatch, the latency to escape the home cage was measured. An animal was considered as escaped when all four paws of the animal were over the edge of the cage, either by climbing onto the grid or by jumping on top of one of the cage walls. The cutoff to escape the home cage was set at 10 min and rats who did not escape were given that score for the latency.

### Corticosterone levels

Animals were socially housed at the time of blood sampling. Blood sampling was performed before the start of the FST and directly after the FST on day 1 (between 12:00 and 17:00) to measure the response to a stressor. Blood was sampled from the experimental animal within 20 min after arriving in the experiment room. To make sure no smell of blood lingered after sampling and testing, spilled blood was cleaned before starting the next trial. For blood collecting, a small tail incision was made with a razor blade and ± 150 μL blood was collected in EDTA coated capillary tubes. Blood samples were centrifuged (3000 rpm) for 10 min at 4 °C. The plasma was stored at − 80 °C. CORT levels were determined by radioimmunoassay (MP Biomedicals, Orangeburg, NY). Samples were analyzed in duplo and measures were averaged afterwards.

### Surgery and telemetry

At 18 weeks, animals were anesthetized with a mixture of N2O/O2 (1:2) and isoflurane (2.5%, Rhodia Organique Fine Limited, Bristol, UK). A temperature-sensitive transmitter (Type TA10-TA-F40, Data Sciences Inc., St. Paul, Minnesota, USA) was implanted in the peritoneal cavity. Immediately after surgery and again after 24 h, animals received 0.2 mL finadyne (MSD, the Netherlands), an anti-inflammatory and pain relieving drug. Animals were individually housed and placed with their home cage on a receiver (model RPC-1 and RLA1020, Data Sciences Inc.). Output signals from the transmitters were transferred via the receivers to a PC-based data acquisition and analysis system (Dataquest ART version 3.11, Data Sciences Inc.). Activity counts and CBT were sampled every 5 min for 10 s on a 24-h basis. Animals were allowed to recover for 14 days before starting behavioral testing.

### Assessment of circadian behavior

**Fig. 1:**
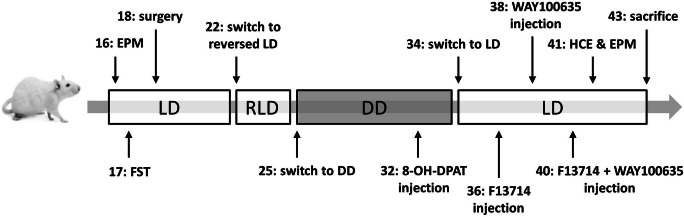
Overview of performed behavioral tests and pharmacological challenges. Lighting conditions: Light-dark (LD), reversed light-dark (RLD), and constant darkness (DD). Numbers represent the age of the animal in weeks

Patterns of activity and body temperature were assessed in three lighting conditions: normal light/dark 12/12 h cycle (LD), reversed light/dark cycle (RLD), and constant darkness (DD) (Fig. 1). From birth until surgery, animals were housed under a 12/12 h LD cycle (lights on at 22:00, off at 10:00, Zeitgeber time (ZT) 0–12). Zeitgeber time (ZT0) was defined as light onset and ZT12 as light offset. At 22 weeks, the LD cycle was reversed by advancing the LD cycle by 12 h (lights on at 10:00, of at 22:00, ZT 12–24). Animals were maintained on this RLD cycle for 3 weeks before changing the cycle to DD for 9 weeks during which the animals received a phase-shifting 8-OH-DPAT injection. Only the last week of RLD data is used for the analysis due to 1 day where the lights were left on accidentally, so a sufficient washout period was left before analyzing the data. After the DD period, animals were put on a normal LD cycle again during which they were exposed to pharmacological challenges of the serotonergic system. Finally, animals were tested for HCE and again in the EPM in this LD cycle, followed by 2 weeks of rest before sacrificing for brain collection.

#### Entrained circadian rhythmicity

Entrainment of activity and core body temperature (CBT) circadian rhythm to a normal 12:12 h LD cycle and RLD cycle was measured during the last 7 days of each period for an average 24 h profile (LD: week 20, RLD: week 24). Activity counts were first z-transformed (and made a positive integer by adding the most negative number in the dataset to all values for that individual) to avoid bias of telemetry sender differs. Subsequently, activity data were averaged over 7 days and grouped into 4 h blocks to determine the average profile over 24 h. Furthermore, the period of the rhythm (tau) of the entrained LD and RLD rhythm was calculated.

#### Circadian response to 8-OH-DPAT

At 32 weeks, all animals received a subcutaneous injection of 5-HT_1A/7_ receptor agonist 8-OH-DPAT (5 mg/kg, Sigma Aldrich Chemie BV, Zwijndrecht, the Netherlands) at a time point where low activity was measured in the DD (ZT 15) to trigger a phase-shifting response. Using the 6 days of the DD period prior to and the day including the 8-OH-DPAT injection, the free-running tau for activity and CBT before 8-OH-DPAT injection (pre-injection) was determined as has been done in a previous study (Kiryanova et al. 2017). In addition, free-running tau for activity and CBT were calculated after 8-OH-DPAT injection (post-injection) using a period of 7 days excluding three transient—that is unstable—cycles after injection (Kiryanova et al. 2017). Subsequently, pre- and post-injection free-running tau were compared and the phase shift was calculated. A periodogram was created for additional visualization of the results.

#### Data analysis

Entrained and free-running tau were calculated by fitting CBT data using locally weighted scatterplot smoothing (R function loess, R Core Team, 2015; version 3.2.3, Rstudio version 0.99.491) to find the timepoint of CBT_min_ for each of the 7 days of data. The time between these CBT_minima_ was then calculated and averaged across the 7-day period. These entrained and free-running tau values were then compared between treatment groups. Entrained and free-running tau for the activity data were calculated using a Lomb-Scargle periodogram analysis (Ruf 1999) performed in Chronoshop (version 1.05, courtesy of Kamiel Spoelstra). The periodogram analysis consisted of a quantitative analysis, with a range of 18 h to 28 h, a resolution set at 2 bins, and an alpha of 0.01. To plot the data, animals were split by treatment group and the normalized power (PN) was plotted for both pre- and post-8-OH-DPAT injection against the tau range included in the analysis. The phase shift was calculated as the time between the two best fitted periods of activity. This analysis was performed in Chronoshop, with a running mean of 10 bins based on center of gravity of the data. One rat (VEH) was excluded from all tau analyses due to a lack of data (battery died in the transponder). Two rats (1 VEH and 1 FLX) had only 5 days instead of 7 days of pre-injection tau data and two other rats had 3 days (FLX) and 5 days (VEH) for the post-injection tau data. An average of the available data was utilized in these cases.

### 5-HT_1A_ receptor sensitivity

#### Thermal response to 5-HT_1A_ receptor agonist F13714

After 8-OH-DPAT analysis, animals were put on a normal 12:12 h LD cycle again (lights off at 07:00) at week 34. After 2 weeks of acclimatization, animals were injected subcutaneously with 0.0625, 0.125, or 0.25 mg/kg of selective 5-HT_1A_ receptor agonist F13714 (Neurocrine biosciences, San Diego, California, USA) or saline (0.9% NaCl) to induce hypothermia (week 36). Recovery was assessed afterwards. Animals received all different concentrations in randomized order with a washout period of 3 days in between injections (within animal design). Injections were given at a time point where low activity was measured (ZT 15).

#### Thermal response to 5-HT_1A_ receptor antagonist WAY100635

After the washout period of F13714, we continued by giving a subcutaneous injection of 5-HT_1A_ receptor antagonist WAY100635 (Wyeth Ayerst Pharmaceuticals, Princeton, New Jersey, USA) in week 38. Animals were injected with 0.001, 0.1, and 1 mg/kg WAY100635 or saline in randomized order and again with a washout period of 3 days in between injections. Injections were given at a time point were low activity was measured (ZT 15).

#### Thermal response to a combination of F13714 and WAY100635

At 40 weeks, a combination of F13714 (0.125 mg/kg, s.c.) and WAY100635 (0.1 mg/kg, s.c.) was given during the low activity period (ZT15), to investigate whether WAY100635 could antagonize the F13714-induced hypothermic response, to confirm the involvement of the 5-HT_1A_ receptor. First animals were injected inside the flank with WAY100635 which was immediately followed by an injection with F13714 in the other flank.

#### Data analysis

For all injections, the area under the curve (AUC) for body temperature was calculated from 1 h before the injection to 4 h after the injection (Olivier et al. 2011).

### Gene expression analysis

#### Sacrificing and brain dissection

After finishing the HCE and EPM in week 41, the beginning of the 12/12 h LD cycle was set at 23:00 lights on for logistic reasons. After 2 weeks of acclimatization to the new cycle, half of the animals (6 VEH, 5 FLX) were sacrificed at the end of the light phase between 8:30 and 9:30 (ZT9.5-CT10.5), while the other half (6 VEH, 6 FLX) was sacrificed at the end of the dark phase between 20:30 and 21:30 (21.5 – CT 22.5). At these time points, the rhythmically expressed clock genes in the SCN that we chose were not at their minimum or maximum expression (Shigeyoshi et al. 1997; Kume et al. 1999) to avoid a floor or ceiling effect that could conceal differences between groups. Animals were anesthetized with CO2 by placement on a grid in a small box directly above dry ice for approximately 60 s, prior to decapitation. Brains were rapidly dissected and frozen in isopentane on dry ice and stored at − 80 °C. Brains were cut into coronal slices (200 μM, − 12 °C) and punches from the SCN (3 slices) were obtained according to the atlas of Paxinos and Watson. Brain punches were obtained bilaterally using a single 1.2 mm punch per slice (Harris Uni-CoreTM) and collected in RNA/DNA-free tubes on dry ice and stored at − 80 °C until further use.

#### RNA isolation, cDNA synthesis, quantitative real-time polymerase chain reaction

Total RNA was isolated from SCN tissue using Trizol reagent (Invitrogen Life Technologies) and stored at − 80 °C until further use. A NanoDrop 2000c spectrophotometer was used to assess RNA concentrations and purity. Complementary DNA (cDNA) was synthesized from RNA using oligo(dT)18 primers through RevertAid Reverse Transcriptase (Thermo Scientific). After cDNA synthesis, samples were diluted to 10 ng/uL cDNA and further processed for qPCR analysis. Using 20 ng of cDNA, mRNA levels of the following genes were assessed: Period 1 (Per1), Period 2 (Per2), Cryptochrome 1 (Cry1), Cryptochrome 2 (Cry2), and the 5-HT_1A_ receptor (5-HT_1A_R). Gapdh and β-actin were used as reference genes for normalization of gene expression. Custom-designed TaqMan® gene expression assays (Thermo Scientific) were used for all genes Per1 (Rn01325256_m1), Per2 (Rn01427704_m1), Cry1 (Rn01503063_m1), Cry2 (Rn01485701_m1), 5-HT_1A_R (Rn00561409_s1), and housekeeping genes Gapdh (RN01775763_g1) and β-actin (RB0667869_m1). qPCR for Gapdh with Per2 and β-actin with Cry2 were performed in multiplex. qPCR for Per1 and Cry1 were performed in singleplex. Quantitative real-time PCR (qRT–PCR) was performed using the CFX96 Real-Time PCR Detection System (Bio-Rad, Hercules, CA, USA). Thermal cycling conditions were as follows: 2 min at 50 °C, 2 min at 90 °C, followed by 50 cycles of 15 s at 95 °C, and 1 min at 60 °C.

#### Data analysis

All samples were run in triplicates, except for 5-HT_1A_R, which was run in duplo due to insufficient amount of Taqman assay. Mean plate efficiencies and Cq values were calculated with the qRT-PCR analysis software LinRegPCR. When the standard deviation between a triplicate was > 0.3, the most outlying sample was removed. For the duplo samples, when the standard deviation was > 0.5, both samples from the animal were removed. Mean normalized expression (MNE), based on the ratio between Cq values of the target and reference genes and the efficiency of the PCR reactions, was calculated as a measure of target gene transcription. Data were presented as logMNE and the fold change was calculated.

### Statistical analysis

First, all data were checked for parametric distribution. Forced swim test immobility and entrained tau in LD and RLD were analyzed using unpaired *t* tests, with treatment as the independent factor. A 2-WAY repeated measures ANOVA was performed for the EPM, with age (16 vs. 41 weeks) as within subjects factor and treatment (VEH vs. FLX) as between subjects factor. CORT levels before and after FST exposure were also analyzed using a 2-WAY repeated measures ANOVA with stress exposure (basal vs. after FST) as within subjects factor, and treatment (VEH vs. FLX) as between subjects factor. For activity and CBT free-running tau pre- and post-injection data, a mixed ANOVA was performed with time as within subjects factor and treatment as between subjects factor. Furthermore, for the 5-HT_1A_ receptor sensitivity data, a mixed ANOVA was performed for the AUC, with dose as within subjects factor and treatment as between subjects factor. Gene expression was analyzed using unpaired *t* test per gene. Data are presented as mean ± SEM and significance was set at *p* < 0.05. When 0.1 < *p* > 0.05, this was considered a tendency.

## Results

### Behavioral testing

#### Elevated plus maze

FLX exposure significantly lowered the entries onto the open arm of the EPM, regardless of testing age (F(1,18) = 6.291, *p* = 0.022). Post hoc testing showed that at 16 weeks of age, before the start of all other experimental procedures, FLX-exposed animals showed fewer entries into the open arm of the EPM than VEH-exposed animals (t(19) = 2.103, *p* = 0.049, VEH *n* = 10, FLX *n* = 11, Table 1). An age of 41 weeks at EPM testing resulted in a lower total distance moved when compared to testing at 16 weeks, regardless of treatment (F(1,18) = 11.104, *p* = 0.004). Post hoc testing showed that FLX-exposed animals had a lower total distance moved in the EPM at 41 weeks of age than at 16 weeks of age (t(9) = 3.215, *p* = 0.011). No other differences between groups were found for the behavioral parameters measured (Table 1).

**Table 1:** Behavioral outcomes in offspring exposed to perinatal FLX or VEH treatment

Behavioral parameter	VEH	FLX	Statistics (unpaired *t* test)
EPM (16 weeks)			
Total distance moved (m)	16.42 ± 0.72	16.39 ± 0.62	n.s.
Time in open arm (sec)	109.51 ± 9.76	115.60 ± 11.11	n.s.
Open arm entries (freq)	12.80 ± 0.93	10.27 ± 0.78	t(19) = 2.103, *p* = 0.049
EPM (41 weeks)			
Total distance moved (m)	15.38 ± 0.57	13.63 ± 0.58	n.s.
Time in open arm (sec)	109.24 ± 9.18	97.30 ± 9.27	n.s.
Open arm entries (freq)	10.67 ± 0.92	8.91 ± 0.64	n.s.
FST			
Immobility time (sec)	99.85 ± 16.55	64.90 ± 8.79	t(22) = 1.866, *p* = 0.075
HCE			
Latency to exit cage (sec)	541.58 ± 39.59	534.91 ± 36.93	n.s.

Data are presented as mean ± SEM

#### Forced swim test

During the 5 min test phase of the FST, FLX-exposed animals tended to spend less time being immobile than VEH-exposed animals (t(22) = 1.866, *p* = 0.075, VEH *N* = 12, FLX *N* = 12, Table 1).

#### Home cage emergence

No differences were found between groups in the latency to escape the home cage (VEH *n* = 12, FLX *n* = 11, Table 1).

### CORT levels

CORT levels significantly increased after FST stress exposure, independent of treatment (F_(1,22)_ = 343,686, *p* < 0.001). Post hoc testing indicated increased CORT levels after FST exposure in both VEH (*t* = − 11.393, df = 11, *p* < 0.001) and FLX (*t* = − 15,458, df = 11, *p* < 0.001) exposed animals. No differences were found in CORT levels between VEH- and FLX-exposed animals at the basal level (VEH 746.2 ng/mL ± 123.8 ng/mL, FLX 748.1 ng/mL ± 109.6 ng/mL), nor after FST stress exposure (VEH 2838.1 ng/mL ± 183.0 ng/mL, FLX 3005.4 ng/mL ± 143.4 ng/mL).

### Circadian behavior

#### Entrained circadian rhythmicity

**Fig. 2:**
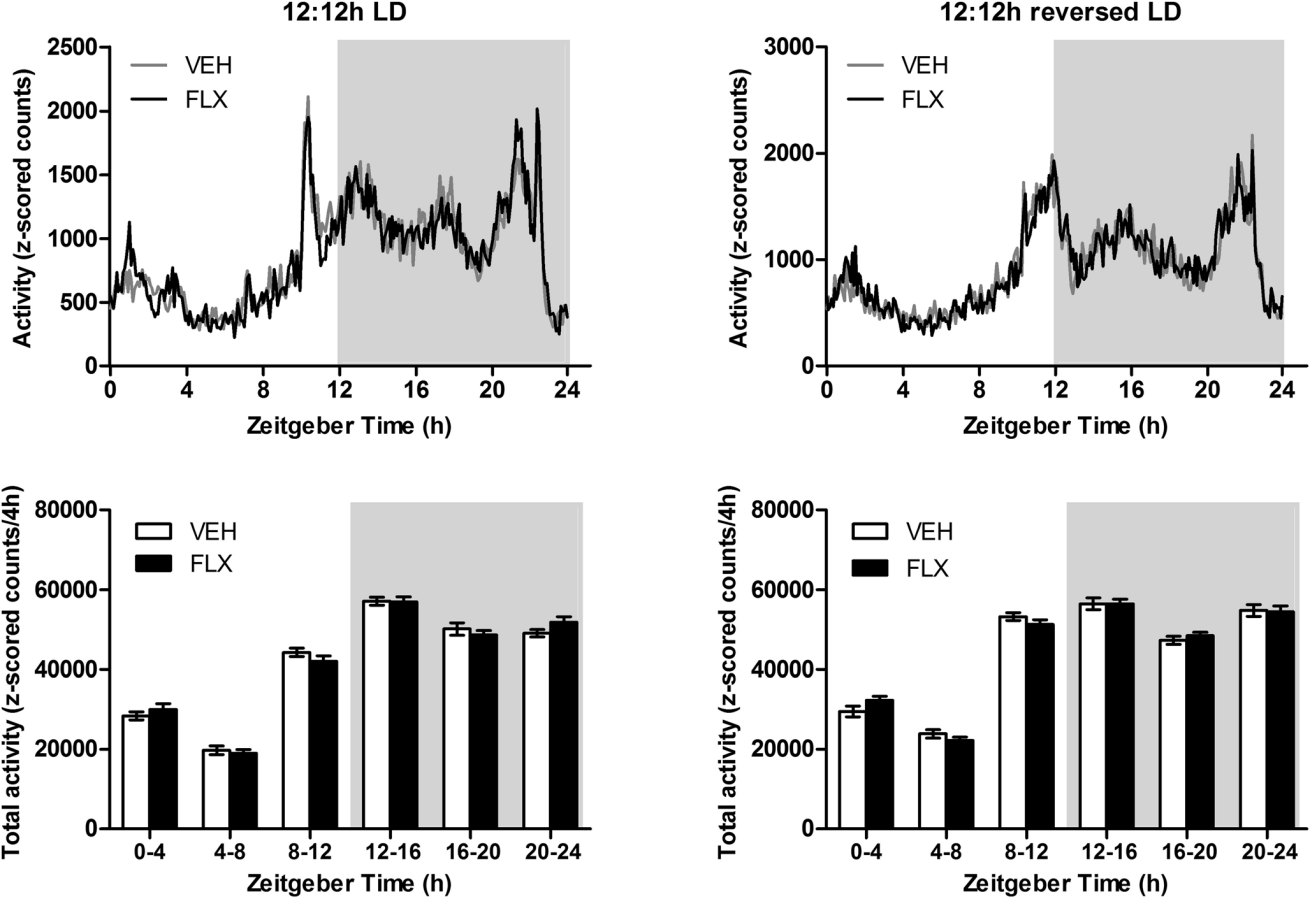
Twenty-four-hour profiles of activity (**a**, **b**) and CBT rhythmicity (**c**, **d**) during the LD (**a**, **c**) and RLD (**b**, **d**) period

The 24 h activity profile indicates similarities between treatment groups in terms of activity level during both the normal light/dark 12/12 h cycle (LD) and reversed light/dark cycle (RLD) cycle. Particularly, when activity was grouped into 4 h periods, no difference in activity was found between VEH- and FLX-exposed animals (Fig. 2a). This lack of difference remained on reversal of the LD cycle (Fig. 2b). Furthermore, the 24 h core body temperature (CBT) profile also did not differ between the two treatment groups during both LD and RLD cycle (Fig. 2c, d). For both CBT and activity, entrained tau was not significantly different between groups (Table 2).

**Table 2:** Entrained and free-running circadian rhythmicity under different light regimes

	Light regime	VEH	FLX	Statistics
Activity				
Entrained tau	LD	23.86 ± 0.06	23.97 ± 0.07	n.s.
	RLD	24.00 ± 0.09	23.89 ± 0.10	n.s.
Free-running tau	DD (pre-8-OH-DPAT injection)	24.00 ± 0.07	24.18 ± 0.08	n.s.
	DD (post-8-OH-DPAT injection)	24.08 ± 0.07	23.91 ± 0.06^1^	t(8) = 2.866, *p* < 0.021
Core body temperature				
Entrained tau	LD	23.87 ± 0.07	23.92 ± 0.05	n.s.
	RLD	24.27 ± 0.04	24.27 ± 0.07	n.s.
Free-running tau	DD (pre-injection)	24.02 ± 0.06	23.85 ± 0.05	n.s.
	DD (post-injection)	24.01 ± 0.07	24.03 ± 0.08	n.s.

1, vs FLX pre-injection; *LD*, normal light/dark cycle; *RLD*, reversed light/dark cycle; *DD*, constant darkness. Data are presented as mean ± SEM

#### Circadian response to 8-OH-DPAT

**Fig. 3:**
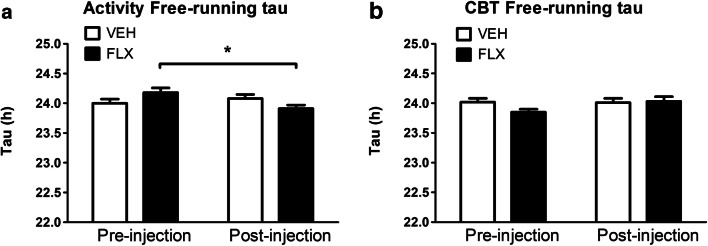
Free-running tau of activity (**a**) and CBT (**b**) pre- and post-8-OH-DPAT injection. *p < .0.05

**Fig. 4:**
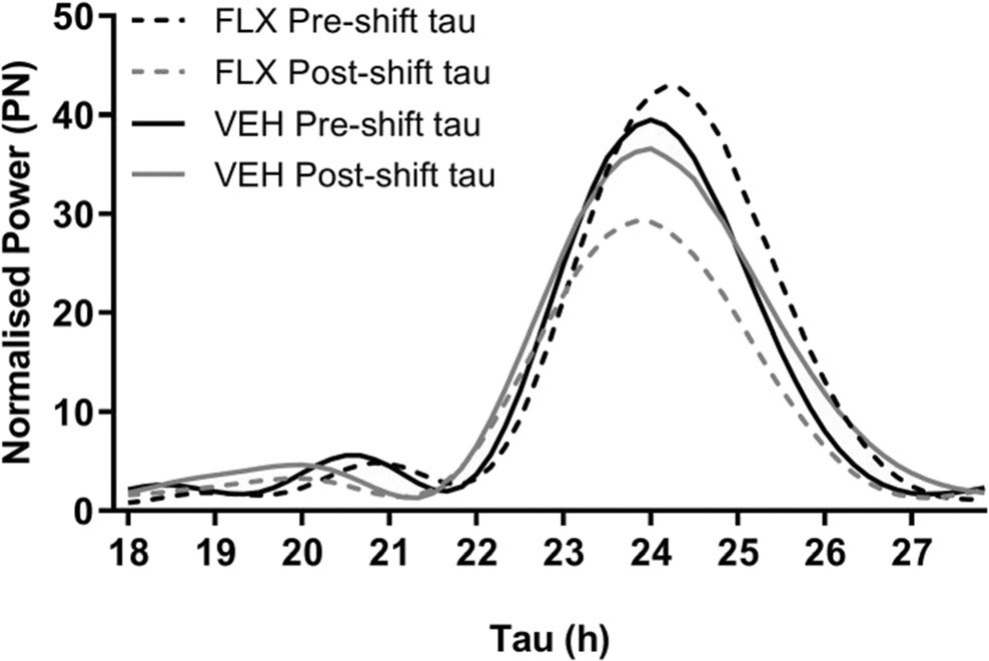
Periodogram visualizing changes in activity free-running tau of VEH and FLX exposed animals pre- and post-8-OH-DPAT injection

FLX treatment and injection time (pre- or post-injection) significantly interacted to affect the free-running tau for activity (F_(1,15)_ = 5.477, *p* = 0.034, Fig. 3a). FLX exposure significantly shortened the free-running tau after the 8-OH-DPAT injection, when compared to before the injection (t_(8)_ = 2.866, *p* = 0.021), while free-running tau of VEH exposed animals was unaffected (Table 2). For additional visualization of these results, a periodogram was made (Fig. 4). No differences between treatments were found in CBT either pre- or post-injection (Table 2) and no main or interaction effects were found for CBT free-running tau (Fig. 3b).

### 5-HT_1A_ receptor sensitivity

#### Thermal response to 5-HT_1A_ receptor agonist F13714

**Fig. 5:**
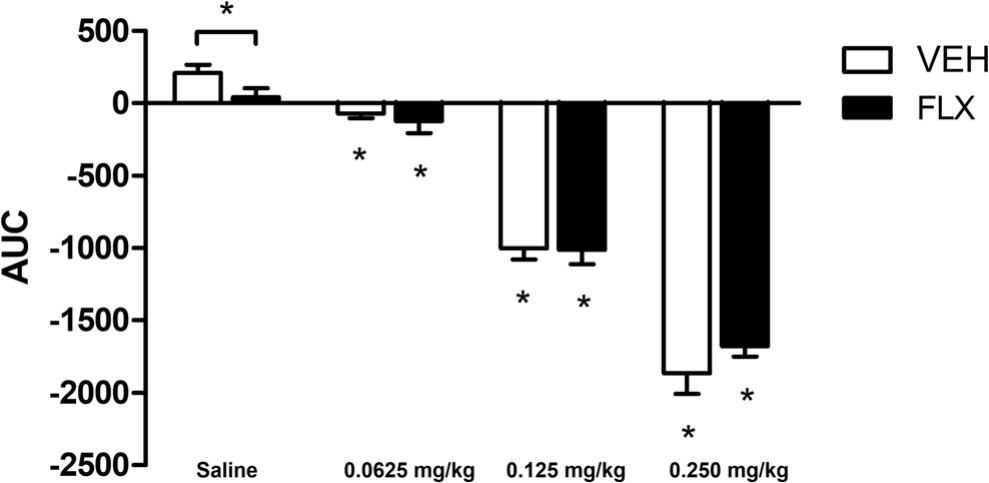
Thermal response to 5-HT_1A_ receptor agonist F13714. *p < 0.05 compared to same-treated (VEH or FLX) saline injected animal.

We investigated 5-HT_1A_ receptor sensitivity by applying a specific and highly efficacious 5-HT_1A_ receptor agonist (F13714) in a dose-response manner and consequently by measuring the hypothermic effect. A significant effect of F12714 dose was found on the area under the curve (AUC) (F_(3, 51)_ = 311.593, *p* < 0.001), and a trend towards a dose x treatment interaction was found (F_(3, 51)_ = 2.205, *p* = 0.099) (Fig. 5). All doses significantly decreased the body temperature in both VEH (0.0626 mg/kg, *p* = 0.001; 0.125 mg/kg, *p* < 0.001; 0.25 mg/kg, *p* < 0.001) and FLX (0.0626, *p* = 0.044 mg/kg; 0.125 mg/kg, *p* < 0.001; 0.25 mg/kg, *p* < 0.001) offspring compared to a saline injection, as indicated by a negative AUC (Fig. 5). In addition, FLX offspring had a significant smaller thermal response to F13714 than VEH offspring after a saline injection (*p* = 0.023).

#### Thermal response to 5-HT_1A_ receptor antagonist WAY100635

When injected with 5-HT_1A_ receptor antagonist WAY100635, an effect of dose on the AUC was found (F_(3,54)_ = 3.171, *p* = 0.031). Post hoc testing revealed that VEH offspring injected with a dose of 0.1 mg/kg WAY100635 had a lower CBT than VEH offspring injected with 0.01 mg/kg (*p* = 0.048, Table 3).

**Table 3:** Thermal response to 5-HT_1A_ receptor agonist F1371, 5-HT_1A_ receptor antagonist WAY100635 and their combination

	Area under curve (AUC)		
	VEH	FLX	Statistics
F13714			
Saline	208.63 ± 57.14	41.37 ± 63.51^*^	*p = 0.023 vs. Saline VEH
0.0625 mg/kg	− 70.66 ± 34.23	− 123.15 ± 80.07	n.s.
0.125 mg/kg	− 1002.43 ± 76.08	− 1012.21 ± 99.23	n.s.
0.250 mg/kg	− 1864.82 ± 141.46	− 1679.31 ± 71.85	n.s.
WAY100635			
Saline	72.32 ± 33.75	81.85 ± 55.73	n.s.
0.01 mg/kg	234.67 ± 68.15	128.38 ± 38.45	n.s.
0.1 mg/kg	68.63 ± 27.46^*^	16.44 ± 59.81	*p = 0.048 vs. 0.01 mg/kg VEH
1 mg/kg	111.19 ± 62.92	108.71 ± 38.35	n.s.
WAY100635 (0.1 mg/kg) + F13714 (0.125 mg/kg)	− 366.16 ± 75.32	− 354.37 ± 100.12	n.s.

Data are presented as mean ± SEM. AUC was calculated from 1 h before the injection to 4 h after the injection. A positive AUC indicates a hyperthermic response, while a negative AUC indicates a hypothermic response

#### Thermal response to a combination of F13714 and WAY100635

Next, a combination of 0.1 mg/kg WAY100635 and 0.125 mg/kg F13714 was administered to the animals. No differences were found in the AUC between VEH- and FLX-exposed animals (Table 3). However, the drop in CBT (negative AUC) was smaller than when F13714 was given alone, suggesting that WAY100635 partially blocks the hypothermia-induced response to F13714.

### Gene expression analysis

SCN gene expression was analyzed for 5-HT_1A_R and four clock genes: Per1, Per2, Cry1, and Cry2. SCN gene expression did not differ between VEH- and FLX-exposed animals for any of the genes analyzed (Table 4).

**Table 4:** SCN gene expression of the 5HT_1A_ receptor and four clock genes

Genes	VEH LogMNE	FLX LogMNE	Fold change
Per1	− 12.18 ± 0.37	− 12.02 ± 0.43	− 0.01
Per2	− 7.15 ± 0.61	− 7.61 ± 0.89	0.07
Cry1	− 10.04 ± 0.51	− 9.67 ± 0.49	− 0.04
Cry2	− 11.59 ± 0.39	− 10.90 ± 0.40	− 0.06
5-HT_1A_R	− 8.79 ± 1.00	− 10.23 ± 0.63	0.06

Data, except for the fold change, are presented as mean logMNE ± SEM

## Discussion

Our findings provide evidence that perinatal FLX treatment disrupts the circadian response to a phase-shifting challenge in female rats. The 5-HT_1A/7_ receptor agonist 8-OH-DPAT shortened the free-running tau for activity in rats that were perinatally exposed to FLX, while this effect was absent in the VEH exposed animals. No differences were found between FLX- and VEH-exposed rats in free-running tau on the CBT before and after injection of 8-OH-DPAT, indicating that only after a challenge the disruption in the circadian response becomes apparent in the activity and not in CBT. In mice, perinatal FLX exposure has also been shown to reduce the free-running period compared to control mice. This effect was already present without an 8-OH-DPAT challenge, although the advanced phase shift caused by the 8-OH-DPAT challenge was smaller in mice that were perinatally exposed to FLX (Kiryanova et al. 2013) which is in line with our findings. As for involved mechanisms, SSRIs have been found to shorten the period of clock gene Per1 in both rat-1 fibroblasts and in the mouse SCN (Nomura et al. 2008). We investigated whether the key clock genes Per1, Per2 and Cry1 and Cry2 were altered due to perinatal FLX exposure but did not find any differences in the expression of these genes in the SCN when compared to VEH exposed animals. There might be several reasons for this finding. First, the expression of period and cryptochrome genes in the adult brain might not be affected by perinatal FLX exposure. Second, only differences in free-running tau for activity were found after the 8-OH-DPAT challenge, and we did not use this challenge before sacrificing the rats. In hamsters, 8-OH-DPAT caused an inhibitory effect on SCN Per1 and Per2 expression (Horikawa et al. 2000). For future studies, it would be worthwhile to investigate whether similar effects would occur in our rats after an 8-OH-DPAT challenge, and whether perinatal FLX exposure alters those inhibitory responses. Third, it has been shown that there is a circadian time-dependent effect caused by 8-OH-DPAT (Horikawa et al. 2000), and even though we sacrificed the animals at two time points (end of light and end of dark phase), we might have missed the time point where differences arise in gene expression. In the study of Horiwaka and colleagues, differences in Per1 and Per 2 expression after an 8-OH-DPAT challenge were only found at the mid-subjective day, and not during the early subjective day or subjective night, indicating that the effect might only be found in a small timeframe (Horikawa et al. 2000). Fourth, we measured mRNA levels which do not necessarily reflect protein levels of the genes. Further research is necessary to see whether protein levels confirm mRNA levels. Altogether, perinatal FLX exposure disrupts the circadian response to a phase-shifting challenge, but underlying mechanisms causing this effect still remain to be investigated.

Because 8-OH-DPAT exerts its phase-shifting effect (partly) via the 5-HT_1A_ receptor (Smith et al. 2008), and because SSRIs are known to change the sensitivity of 5-HT receptors (Olivier et al. 2011), we investigated whether 5-HT_1A_R expression in the SCN and whether the sensitivity to a 5-HT_1A_ receptor agonists on hypothermia was changed due to perinatal FLX exposure. No differences were found in the 5-HT_1A_R expression in the SCN. SSRIs are known to influence the sensitivity of 5-HT receptors including the 5-HT_1A_ receptor. Here, we showed that 5-HT_1A_R expression in the SCN is not altered due to perinatal FLX exposure, although we did not test the final 5-HT_1A_ receptor protein levels. On a functional level, we tested the responsivity to the 5-HT_1A_ receptor agonist F13714 on hypothermia. A clear dose-response effect was found in both VEH and FLX exposed rats; however, no differences in the hypothermic response were found. These findings indicate that the functionality of the 5-HT_1A_ receptor is not altered in female rats after perinatal FLX exposure, at least not those 5-HT_1A_ receptor populations involved in the hypothermic response. In previous experiments, we showed that perinatal FLX exposure slightly increased the sensitivity of the 5-HT_1A_ receptor to flesinoxan-induced hypothermia, albeit in male rats (Olivier et al. 2011). Therefore, the discrepancy between the current and previous study might be explained by sex differences. Interestingly, we found that stress-induced hyperthermia (SIH) is reduced in female rats that were perinatally exposed to FLX. A similar effect was found in SERT^−/−^ rats. After a saline injection, SERT^−/−^ rats showed a reduced SIH, indicating that high extracellular levels of 5-HT during development cause a shift in the sensitivity to this stressor (Olivier et al. 2008). A relative hyperstimulation of 5-HT_1A_ receptors, due to FLX exposure, might underlie the decrease in stress-induced hyperthermia, as this paradigm is mediated by the 5-HT_1A_ receptor (Olivier et al. 1998). However, further research is necessary to investigate whether the sensitivity of 5-HT_1A_ receptors implicated in SIH is reduced after perinatal FLX exposure.

Finally, we investigated the behavioral outcome in female rats that were perinatally exposed to FLX. Our data show that perinatal FLX exposure does not induce anxiety-like behavior in female rats. Animals were tested twice in the elevated plus maze (EPM), once before all other experiments, and once at the end after all experiments. This was done because we previously showed higher anxiety levels in the EPM after a stressful experiment (conditioned place aversion with shock) compared to before this experiment (Olivier et al. 2011). In the current study, no robust increase in anxiety levels in the EPM was found, If any, a decreased number of entries on the open arm in the first EPM experiment, indicating a slight increase in anxiety levels. Reasons for the discrepancy with the previous study might be that we used females in the current study as opposed to males. In addition, different stressors were used in both studies, making the comparison more challenging. Nevertheless, our study suggests that perinatal FLX exposure in female rats does not profoundly increase anxiety levels since the open arm time is similar to the VEH exposed animals, which is in line with other studies in both male and female rodents (Ansorge et al. 2004, 2008; Lisboa et al. 2007; Olivier et al. 2011; Ko et al. 2014; Altieri et al. 2015; Silva et al. 2018). The fact that perinatal FLX exposure does not increase anxiety levels in female rats was also confirmed in our home cage emergence test, where no difference in latency to escape was found between FLX and VEH exposed animals. To assess the effects of perinatal FLX exposure on stress coping, immobility time was assessed in the forced swim test. No effects of perinatal FLX exposure on immobility in the FST were found, which agrees with another study in females (Altieri et al. 2015). However, increased immobility time (Lisboa et al. 2007; Ko et al. 2014; Rebello et al. 2014; Boulle et al. 2016b), but also decreased immobility time (Karpova et al. 2009) has been found in the forced swim test after developmental FLX exposure. It is hard to compare all these results as experimental design, sex of animals, and exposure timing all vary in these studies. Finally, basal CORT levels were measured before and after the swim test, to investigate whether the stress response was altered due to perinatal FLX exposure. CORT levels did increase after the forced swim test in both VEH- and FLX-exposed animals; however, no differences were found between groups. A previous study in rats showed that FLX exposure during the early postnatal period results in lower basal serum CORT levels in male, but not female, adolescents (Pawluski et al. 2012). In female mice, prenatal FLX exposure results in a greater CORT response to acute restraint stress (Avitsur 2017). The fact that we did not find any differences in basal and stress response CORT levels between our VEH and FLX exposed animals might be explained by the stressor we used. The forced swim test appeared to be highly stressful as CORT levels after the test were extremely high, which may have caused a ceiling effect. Therefore, using a more commonly used (milder) stressor, e.g., restraint stress, to measure CORT levels would be more appropriate in future studies.

In conclusion, perinatal FLX treatment in female rats did not have a major effect on anxiety-like behavior, stress coping, and 5-HT_1A_ receptor sensitivity. In addition, circadian rhythmicity seems largely intact. The only significant observation found in the present study was the shortened free-running tau followed by a nonphotic challenge in perinatal FLX-treated rats compared to VEH- treated rats. Whether this altered response contributes to vulnerability of health risks remains to be investigated. Overall, results indicate that female rats may be resilient to the effects of FLX exposure during pregnancy and the postnatal period. Testing females is therefore highly important as findings might deviate from findings found in males.
